# Accuracy of gross tumour volume delineation with [68Ga]-PSMA-PET compared to histopathology for high-risk prostate cancer

**DOI:** 10.2340/1651-226X.2024.39041

**Published:** 2024-06-23

**Authors:** Maryam Zarei, Elin Wallsten, Josefine Grefve, Karin Söderkvist, Adalsteinn Gunnlaugsson, Kristina Sandgren, Joakim Jonsson, Angsana Keeratijarut Lindberg, Erik Nilsson, Anders Bergh, Björn Zackrisson, Mathieu Moreau, Camilla Thellenberg Karlsson, Lars. E. Olsson, Anders Widmark, Katrine Riklund, Lennart Blomqvist, Vibeke Berg Loegager, Jan Axelsson, Sara N. Strandberg, Tufve Nyholm

**Affiliations:** a Department of Diagnostics and Intervention, Biomedical Engineering and Radiation Physics, Umeå University, Umeå, Sweden; b Department of Diagnostics and Intervention, Oncology, Umeå University, Umeå, Sweden; c Department of Hematology, Oncology and Radiation Physics, Skane University Hospital, Lund, Sweden; d Department of Diagnostics and Intervention, Diagnostic Radiology, Umeå University, Umeå, Sweden; e Department of Medical Biosciences, Pathology, Umeå University, Umeå, Sweden; f Department of Translational Medicine, Medical Radiation Physics, Lund University, Malmö, Sweden; g Department of Molecular Medicine and Surgery, Karolinska Institutet, Solna, Sweden; h Department of Radiology, Copenhagen University Hospital in Herlev, Herlev, Denmark

**Keywords:** Prostate cancer, PSMA-PET, histopathology, semi-automatic segmentation

## Abstract

**Background:**

The delineation of intraprostatic lesions is vital for correct delivery of focal radiotherapy boost in patients with prostate cancer (PC). Errors in the delineation could translate into reduced tumour control and potentially increase the side effects. The purpose of this study is to compare PET-based delineation methods with histopathology.

**Materials and methods:**

The study population consisted of 15 patients with confirmed high-risk PC intended for prostatectomy. [^68^Ga]-PSMA-PET/MR was performed prior to surgery. Prostate lesions identified in histopathology were transferred to the in vivo [^68^Ga]-PSMA-PET/MR coordinate system. Four radiation oncologists manually delineated intraprostatic lesions based on PET data. Various semi-automatic segmentation methods were employed, including absolute and relative thresholds, adaptive threshold, and multi-level Otsu threshold.

**Results:**

The gross tumour volumes (GTVs) delineated by the oncologists showed a moderate level of interobserver agreement with Dice similarity coefficient (DSC) of 0.68. In comparison with histopathology, manual delineations exhibited the highest median DSC and the lowest false discovery rate (FDR) among all approaches. Among semi-automatic approaches, GTVs generated using standardized uptake value (SUV) thresholds above 4 (SUV > 4) demonstrated the highest median DSC (0.41), with 0.51 median lesion coverage ratio, FDR of 0.66 and the 95th percentile of the Hausdorff distance (HD95%) of 8.22 mm.

**Interpretation:**

Manual delineations showed a moderate level of interobserver agreement. Compared to histopathology, manual delineations and SUV > 4 exhibited the highest DSC and the lowest HD95% values. The methods that resulted in a high lesion coverage were associated with a large overestimation of the size of the lesions.

## Introduction

External beam radiotherapy plays an important role in the management of localized prostate cancer (PC). Relapses after conventional radiation therapy have been shown to occur primarily at the site of the dominant intraprostatic lesions [1, 2]. Therefore, focal intraprostatic boost was proposed as a method of dose escalation without increasing the dose to adjacent organs at risk, such as the rectum or the urethra. The concept has been demonstrated in clinical trials [3, 4]. In order to balance the benefits of higher dose to the tumour with the risk for toxicity, it is of importance that the definition of the tumour region is as accurate as possible.

The delineated intraprostatic lesions have, in many reports, been obtained by using multi-parametric magnetic resonance imaging (mpMRI) [5, 6]. Additionally, prostate-specific membrane antigen positron-emission tomography (PSMA-PET) has emerged as a valuable clinical tool for imaging of clinically significant PC [7, 8]. PSMA-PET has been reported beneficial in initial staging of high-risk PC and in restaging of recurrent PC [9]. Several studies have validated PSMA-PET-based contouring techniques for intraprostatic lesions by comparing results with histopathology as the standard reference. However, in these studies, either the analysis was based on dividing the prostate in each computed tomography (CT) slice into four equal quadrants [10, 11] or focused solely on investigating dominant intraprostatic lesions within the mid-gland, excluding those in the apex and base [12]. The contours in these studies were delineated manually or derived using a specific percentage threshold of intraprostatic standardized uptake value (SUV) maximum value.

Various automatic or semi-automatic methods have been proposed for estimation of the gross tumour volume (GTV) based on PET data, such as threshold-based methods, gradient-based methods, active contour methods, and classifier-based approaches [13, 14]. Semi-automatic methods such as adaptive thresholding and multi-level Otsu thresholding have not been thoroughly investigated for PSMA-PET-based GTV delineation. The goal of the present study was to compare manual delineations and different PET-based semi-automatic segmentation methods including absolute and relative thresholds, adaptive thresholding, and multi-level Otsu thresholding with areas confirmed by histopathology showing Gleason grades 4 and 5 in high-risk PC patients.

## Materials and methods

### Patient population

The research population in this study is a subset of the participants from PSMA, Acetate and Multiparametric MRI for Prostate cancer (PAMP) dataset, which has ethical approval (Dnr 2016-220-31M) [15]. The study participants had intermediate to high-risk prostate cancer and were planned for laparoscopic radical prostatectomy at Umea University Hospital. The inclusion criteria were as follows: signed informed consent, age over 18 years, Gleason score ≥ 3+4, and an interval of at least 2 months since last prostate biopsy for tissue healing as biopsy procedures may lead to bleeding and temporary inflammation at the biopsy site.

The current study concentrated on high-risk prostate cancer patients with at least one histopathologic slice within the dominant intraprostatic lesion with Gleason score ≥ 4+4 and radiologically identified intraprostatic lesion on either mpMRI or PSMA-PET (or both) as additional inclusion criteria. Of the 55 study participants in PAMP dataset, 15 met the specific inclusion criteria and were included in the present analysis. Supplementary Table S1 summarizes data regarding the study population characteristics.

### PET/MRI acquisition

Data acquisition was performed using a GE Healthcare SIGNA PET/MR 3T scanner (Milwaukee, WI, USA), with an average scan start 61 min post injection. PET data were reconstructed with the Sharp IR reconstruction, which is an ordered subsets expectation maximization (OSEM) technique with point spread function (PSF) modelling, employing 3 iterations, 28 subsets, 3 mm Gaussian post-filtering. The reconstructed images had voxel size of 2.34 × 2.34 × 2.78 mm^3^. The MRI protocol was a diagnostic multiparametric pelvic protocol, including morphological three-plane T2-weighted (T2W) sequences (transaxial, coronal, and sagittal). Detailed information about the sequence parameters is tabulated in Supplementary Table S2.

### Histopathology reference standard

The lesion annotations based on the whole-mount histopathology examination were transferred to the coordinate system of the in-vivo PET/MR examination using the workflow described by Sandgren et al. [16]. The registration of each histopathology slice with its corresponding ex-vivo MRI slice was achieved through 2D-affine registration. The ex-vivo MRI was registered to the in-vivo Axial T2W MRI using a 3D-rigid registration, and the resulting transform was applied to the histopathology stack. Moreover, a deformable registration process with contour correction was conducted to address any potential distortion of the specimen occurring before or during its placement inside the prostate mold. A median in-plane error of 1.7 mm was reported for the registration process [16]. The simultaneous acquisition of these two modalities led to an inherent registration of MRI with PET, which gave a stable foundation for registering histology to PET data. The entire registration process was performed in MICE toolkit (Nonpi Medical AB, Umeå, Sweden) [17], which employs the Elastix image registration software program [18], based on the Insight Toolkit code [19]. Gleason grade 4 and 5 regions were included in the further calculations.

### GTV delineation approaches

To compare different PSMA-PET-based delineation methods with histopathology data, PET images were trimmed to include only the prostate with a 2-pixel margin, based on the T2W MR images. Subsequently, a manual correction was applied to remove portions that included the bladder. This pre-processing was employed only for semi-automatic segmentation methods. The segmentation function applied for adaptive threshold was sourced from the MATLAB Image Processing Toolbox (R2023a). Remaining image analysis was done using Hero (Hero imaging AB, Umeå, Sweden).

### Manual GTV contouring of intraprostatic lesions

Initially, the manual GTV contouring of intraprostatic lesions was performed independently for each case by four radiation oncologists from two different treatment centres, each with more than 10 years of experience as radiation oncologists. The instructions were to create a GTV for focal boosting. Both a radiological report and anatomical imaging with T2W-MRI were available for each patient, but the histopathology data was blinded. Annotations were conducted within a larger study comparing GTV delineations on mpMRI and PET with histopathology to formulate a manual GTV delineation recommendation. Radiation oncologists were permitted to review all image series (T2W, diffusion-weighted, dynamic contrast-enhanced MR, and PET) before delineation. Instructions emphasized considering only visible information in the delineating image, prohibiting fusion of images from different modalities or switching between them. A window level of 0–10 SUV displayed in grey scale was applied to the PSMA-PET data. The treatment planning system used for the delineations was Oncentra (Elekta AB, Stockholm, Sweden) and one observer used Eclipse (Varian Medical System, Palo Alto, CA). In the next step, the delineated structures were imported to Hero for the further comparisons. Both the individual delineations and consensus delineations using the simultaneous truth and performance level estimation (STAPLE) [20] were compared with the histopathology. The resulting GTV, derived from the STAPLE algorithm, is referred to as GTV_manual_ in the following sections of this paper.

### Absolute and relative threshold methods

In the absolute threshold method, SUV threshold of 2, 2.5, 3, 3.5, 4, and 4.5 were used. Pixels with intensities exceeding the threshold were classified as tumour while those below the threshold were categorized as background. The GTVs obtained using these thresholds were referred to as GTV_2–4.5_. The relative threshold method used a threshold calculated as a certain percentage of the maximum SUV value (SUV_max_) for the region of interest. In our study, a threshold range of 20%, 30%, 40% and 50% of SUV_max_ was examined. The generated GTVs were called GTV_20%–50%_. These threshold methods utilized connected components. For instance, the maximum SUV values were selected from the entire pre-processed image (with the prostate within a two-pixel margin and the bladder removed).

### Adaptive threshold

In order to distinguish tumour and background pixels in the images, the adaptthresh function from MATLAB calculates the adaptive threshold value for each pixel depending on the local mean intensity. A parameter *S* (called *Sensitivity* in the toolbox) is a user-adjustable parameter that indicates the sensitivity to thresholding additional pixels as tumour [21]. Initially, values of *S* ranging from 0 to 0.5 were evaluated. However, since those exceeding *S* = 0 had no effect on the study’s conclusion, they were reported in Supplementary Table S3. Consequently, only the adaptive thresholding method with the lowest *S* value (*S* = 0) was reported, and the resulting GTV from this method was referred to as GTV_adaptive_.

### Multi-level Otsu threshold

The basic Otsu approach assumes that there are two separate pixel classes present in an image, such as the foreground and background, and then calculates the optimum threshold to distinguish between these two classes of pixels. Multi-level Otsu thresholding is an expansion of the basic Otsu thresholding method used for image segmentation. Multi-level Otsu aims to divide an image into multiple classes according to the pixel intensities and it calculates the threshold by maximising the variation between the different classes while minimising the variance within each class [22]. In this study, two methods were employed: one involved setting the number of thresholds to two (Otsu2), resulting in three classes, and the other utilized three thresholds (Otsu3), creating four classes in the image. The class with the highest intensity values was chosen as the GTV for each threshold value, referred to as GTV_Otsu2_ and GTV_Otsu3_.

### Statistical analysis

In this study, volumetric comparisons were done between imaging findings and whole-mount histopathology with detailed delineations of Gleason grade 4 and 5. The interobserver agreement for manual delineations was assessed using the Dice similarity coefficient (DSC), referred to as DSC_Interobserver_ [23]. With considering histopathology as the reference, the DSC and the 95th percentile of the Hausdorff distance (HD95%) were calculated for each generated volume produced by the methods. Additionally, the lesion coverage ratio, representing the proportion of histopathology lesion covered by each method, was computed. The false discovery rate (FDR) was determined by calculating the ratio of false positive volume (with considering histopathology as the reference) to the entire volume obtained from each method. Furthermore, the ratio of the GTV of each method to the histopathology volume was calculated as GTV/Histopathology volume ratio.

For each method and lesion, one measure was obtained, and the median values and interquartile ranges for the DSC, HD95%, lesion coverage ratio, FDR and GTV/Histopathology volume ratio were calculated to summarize the results. Gleason Grade 3 regions were considered neither positive nor negative. The Wilcoxon signed-rank test was used for comparison of the measured volumes between methods. All statistical analyses were performed using Statistical Package for the Social Sciences (SPSS v28.0, IBM Corp, NY, USA) with the significance level set at 0.05.

## Results

**Figure 1:**
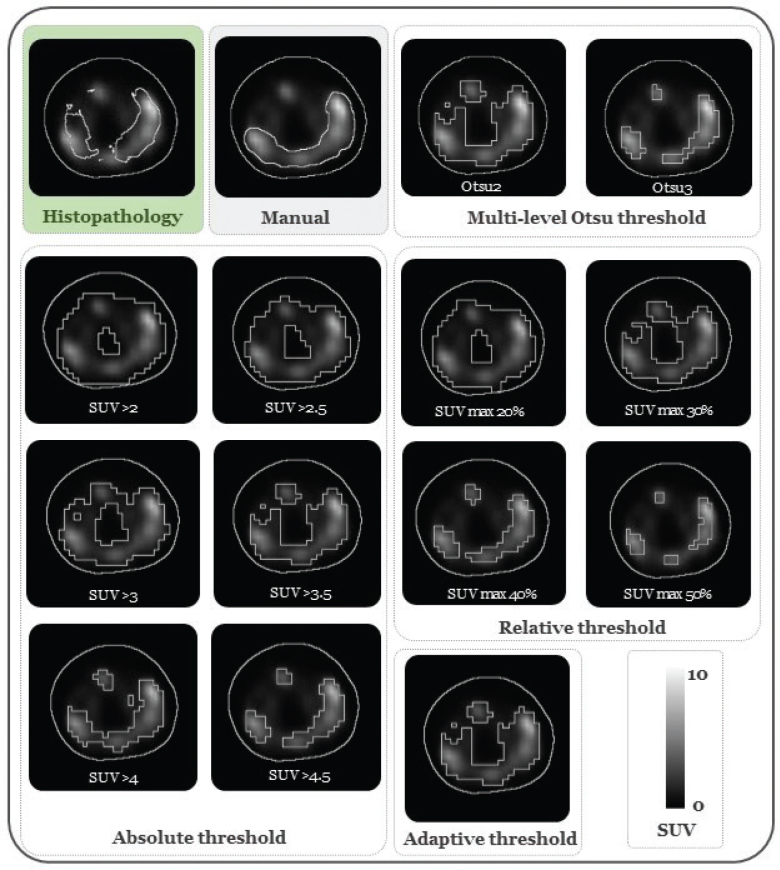
An example of intraprostatic lesion delineation using histopathology, manual delineations, and other semi-automatic segmentation methods in the whole prostate. All the histopathology areas in the figure belong to the same lesion and were determined from the z-direction evaluations of the histopathology data. PET images scaling is SUV min-max: 0–10.

Figure 1 illustrates the delineations of intraprostatic lesion areas obtained from histopathology data and various PET-based approaches for one patient. The GTVs delineated by the four oncologists showed a moderate level of DSC_Interobserver_ (0.68). Supplementary Table S4 provides detailed information about the lesion coverage of each oncologist’s GTV and the unified GTV after applying the STAPLE algorithm, along with the median measured volumes. The median GTV_manual_ was 1.30 ml (0.82–2.28) and demonstrated no statistically significant differences (*p* > 0.05) compared to the volumes measured in histopathology of 1.64 ml (0.46–2.48). Compared to histopathology, GTV_manual_ (after using the STAPLE algorithm [20]) had median lesion coverage ratio of 0.47 (0.21–0.67), DSC of 0.44 (0.24–0.58), FDR of 0.55 (0.32–0.80) and median HD95% of 7.58 mm.

The GTVs obtained using lower SUV thresholds, such as GTV_2_, exhibited high median lesion coverage ratio (0.93) and HD95% (21.96 mm) values. However, these volumes also demonstrated low DSC and high FDR. Among all the semi-automatic methods, GTV_4_ showed the highest median DSC value of 0.41 and the lowest median HD95% value (8.22 mm). GTV_4_ resulted in median lesion coverage ratio of 0.51 with FDR of 0.66. Among the thresholding methods using percentage of SUV_max_ values, GTV_20%_ and GTV_30%_ showed relatively similar median and interquartile ranges in DSC values. However, GTV_20%_ exhibited higher lesion coverage ratio, FDR and HD95% values. The GTV_4.5,_ GTV_40%,_ GTV_50%_ showed no significant difference (*p* > 0.05) compared to the volume measured in histopathology. The adaptive threshold yielded a median lesion coverage ratio of 0.78, a DSC of 0.29, an FDR of 0.82, and HD95% of 18.31 mm. The GTV_adaptive_ was statistically significantly larger than the volume measured in histopathology (*p* < 0.05). The median lesion coverage ratio calculated of GTV_Otsu2_ was calculated to be 0.61 (0.45–0.85), the DSC was 0.38 (0.10–0.52), the FDR was 0.75 (0.44–0.92) and the HD95% was 10.53 mm. Furthermore, GTV_Otsu2_ was statistically larger than the volumes measured in histopathology (*p* > 0.05). In GTV_Otsu3_, the median lesion coverage ratio was decreased to 0.29 (0.23–0.55), the median DSC of this method was 0.35 (0.08–0.5), FDR was 0.58 (0.41–0.85) and HD95% was 8.50 mm. Table 1 summarises the detailed information about the GTV/Histopathology volume ratio, lesion coverage ratio, DSC, FDR and HD95% values of each method.

**Table 1:** Overview of manual delineations, and various PET-based semi-automatic segmentation methods.

	Median GTV/Histopathology volume ratio (IQR)	Median Lesion coverage ratio (IQR)	Median DSC (IQR)	Median FDR (IQR)	Median HD 95% in mm (IQR)
*Manual*	*1.08 (0.68–2.74)	0.47 (0.21–0.67)	0.44 (0.24–0.58)	0.55 (0.32–0.80)	7.58 (5.46–9.22)
*SUV > 2*	10.39 (3.93–22.36)	0.93 (0.86–0.99)	0.15 (0.07–0.35)	0.92 (0.78–0.96)	21.96 (12.41–27.52)
*SUV > 2.5*	7.62 (3.08–11.77)	0.83 (0.72–0.93)	0.16 (0.10–0.40)	0.91 (0.73–0.94)	18.89 (11.03–25.11)
*SUV > 3*	4.17 (2.48–7.24)	0.72 (0.58–0.89)	0.26 (0.08–0.42)	0.84 (0.70–0.93)	15.83 (8.63–22.53)
*SUV > 3.5*	2.51 (1.75–4.36)	0.61 (0.40–0.82)	0.36 (0.09–0.51)	0.77 (0.59–0.94)	10.60 (6.97–16.41)
*SUV > 4*	1.49 (1.22–3.34)	0.51 (0.23–0.74)	0.41 (0.14–0.54)	0.66 (0.52–0.91)	8.22 (6.55–11.83)
*SUV > 4.5*	*1.27 (0.92–2.82)	0.44 (0.21–0.65)	0.38 (0.12–0.55)	0.65 (0.48–0.85)	8.45 (6.82–11.92)
*SUVmax20%*	3.63 (2.09–9.60)	0.87 (0.62–0.97)	0.38 (0.11–0.54)	0.75 (0.57–0.93)	11.07 (7.75–23.68)
*SUVmax30%*	1.90 (1.01–5.44)	0.64 (0.44–0.73)	0.38 (0.10–0.50)	0.67 (0.44–0.92)	8.45 (6.56–14.81)
*SUVmax40%*	*0.93 (0.57–2.79)	0.38 (0.24–0.62)	0.35 (0.10–0.46)	0.59 (0.36–0.91)	8.57 (6.28–13.85)
*SUVmax50%*	0.60 (0.38–1.31)	0.24 (0.13–0.40)	0.29 (0.09–0.43)	0.57 (0.36–0.90)	10.76 (6.76–16.20)
*Adaptive*	4.46 (2.75–10.28)	0.78 (0.62–0.94)	0.29 (0.08–0.40)	0.82 (0.71–0.94)	18.31 (11.15–23.03)
*Otsu2*	1.82 (1.28–5.85)	0.61 (0.45–0.85)	0.38 (0.10–0.52)	0.75 (0.44–0.92)	10.53 (6.29–13.81)
*Otsu3*	*0.84 (0.65–1.96)	0.29 (0.23–0.55)	0.35 (0.08–0.5)	0.58 (0.41–0.85)	8.50 (5.17–12.32)

Stars (*) represent no statistical significance between the volumes measured in different methods and volumes measured in histopathology, using the Wilcoxon signed-rank test.

## Discussion

In this study, we evaluated different approaches for the segmentation of the dominant intraprostatic lesion based on PET data in patients with high-risk PC, with histopathology (Gleason Grade 4 and 5). As mentioned in previous literature [24, 25], the segmentation process based on PET images faces several challenges, such as noise and resolution-related issues. Manual delineation is often considered the most intuitive method for extracting GTVs from PSMA-PET images. Zamboglou et al. found moderate agreement (DSC_Interobserver_ = 0.56) among experienced teams using different PET image scaling techniques. They also reported a high interobserver agreement (DSC = 0.80) for three teams with different experience levels when using the same windowing level. They stated that having the same PET image scaling technique is effective in achieving comparable and consistent manner even among readers with varying levels of experience [10]. In our study, a consistent window level was used among all oncologists, resulting in DSC_Interobserver_ of 0.68. The differences between our interobserver agreement results (DSC_Interobserver_ = 0.68) and their findings (DSC = 0.80) of three teams with the same windowing technique may come from the fact that in our investigation, four individual oncologists were employed for manual delineations, whereas they had three teams of delineators, each consisting of two members. Additionally, their research included CT scans for anatomical orientation. While manual delineations remain a reasonable option, they might be susceptible to subjectivity [26, 27].

In our data, while achieving median lesion coverage ratio of more than 0.80 in few methods such as SUV > 2 and SUV > 2.5, these methods exhibited relatively low DSC values and high FDR (more than 0.90) with relatively large HD95% (more than 18 mm). This is attributed to volume overestimation, potentially resulting in increased toxicity to normal tissues if treated with focal boost radiotherapy. While threshold value of SUV > 4 exhibited the highest median DSC and the lowest median HD95% values among semi-automatic segmentation methods and generated GTV in all patients, there was one patient for whom the created volume was located completely differently from the histopathology volume, resulting in zero lesion coverage and DSC for that patient. At a threshold value greater than SUV 4.5, no GTV was generated for that patient by the method. It is previously known that partial volume effects can reduce the SUV, especially in small volumes [28, 29]. Therefore, in the context of applying semi-automatic methods for intraprostatic lesion segmentation, especially for patients with low SUV values, manual decisions are still necessary as a final step in clinical settings.

Zamboglou et al. [10] suggested that lower SUV_max_ threshold values, such as 20%, were suitable for PSMA-PET-based focal therapy, primarily due to their exceptional lesion coverage (called sensitivity in their paper), and higher threshold percentages (40% and 50%) could be useful for biopsy guidance. It is worth noting that, in terms of lesion coverage calculations, they divided the prostate into four equal segments on each CT slice. Here, for SUV_max20%,_ the median lesion coverage ratio of 0.87 (0.62–0.97) falls below theirs (1.00), potentially attributable to an entirely different analysis approach.

The results regarding the adaptive threshold resulted in a high lesion coverage but with a large overestimation of the size of the lesions (DSC 0.29, HD95% 18.31 mm). This outcome is expected considering the algorithm’s reliance on the local mean intensity of the PSMA-PET image, while [^68^Ga]-based PSMA radiotracers are observed to exhibit a relatively moderate tumour-to-background signal intensity ratio [30]. The local mean intensity is usually calculated by averaging the signal intensity within a specific region. In cases where the tumour-to-background signal intensity ratio is not high, the algorithm may face some challenges in accurately determining the local mean intensity due to the low contrast between the tumour and the background.

In our dataset, the calculated DSC from GTV_Otsu2_ and GTV_Otsu3_ were 0.38 and 0.35 respectively. However, GTV_Otsu2_ showed much higher lesion coverage ratio compared to the GTV_Otsu3_. This elevated value came with the cost of higher FDR and HD95% values for GTV_Otsu2_. Therefore, one could conclude that, to ensure accurate tumour targeting, Otsu2 could be a reasonable option, whereas for preserving normal tissue, Otsu3 performs better. To the best of our knowledge, this study is the first instance of using the multi-level Otsu threshold for intraprostatic lesion delineation while considering histopathology as the reference standard.

The reconstruction algorithm employed in this study was Sharp IR, which is an OSEM technique with PSF modelling. PSF modelling leads to higher spatial resolution but may also result in the overestimation of lesion activity, especially in small lesions [31]. This affects the measured SUV and can influence the delineation methods.

The uncertainty in registration between imaging and histopathology may impact the numbers presented in Table 1. As a part of the study, we performed an estimation of the impact of the registration uncertainty using a second registration based on the outline of the lesions delineated on MR. The results are presented in the Supplementary Table S5 but did not change the conclusions. Our study had a relatively small sample size, which can limit the generalizability of our findings. Additionally, the use of some methods, such as adaptive threshold and multi-level Otsu, which have not been previously applied in this specific context (PSMA-PET image segmentation compared with histopathology), prevents direct comparisons with existing literature. Further investigations with larger sample size are required to evaluate the performance of these methods.

## Conclusion

Accurate delineation of high-grade areas in PC patients is challenging, even using state-of-the-art imaging technology such as PSMA-PET. Manual delineations from four radiation oncologists showed a moderate level of interobserver agreement. Compared with the histopathology volume, GTV_manual_ showed the median DSC of only 0.44 and the median HD95% was 7.58 mm. The SUV > 4 threshold showed the highest DSC and the lowest HD95% values among the applied semi-automatic segmentation methods. The methods that resulted in a high lesion coverage were associated with a large overestimation of the size of the lesions.

## Supplementary Material

Accuracy of gross tumour volume delineation with [68Ga]-PSMA-PET compared to histopathology for high-risk prostate cancer

## Data Availability

The data that support the findings of this study are available from the corresponding author upon reasonable request.

## References

[1] Pucar D, Hricak H, Shukla-Dave A, Kuroiwa K, Drobnjak M, Eastham J, et al. Clinically significant prostate cancer local recurrence after radiation therapy occurs at the site of primary tumor: magnetic resonance imaging and step-section pathology evidence. Int J Radiat Oncol Biol Phys. 2007;69(1):62–9. 10.1016/j.ijrobp.2007.03.065 17707266

[2] Arrayeh E, Westphalen AC, Kurhanewicz J, Roach III M, Jung AJ, Carroll PR, et al. Does local recurrence of prostate cancer after radiation therapy occur at the site of primary tumor? Results of a longitudinal MRI and MRSI study. Int J Radiat Oncol Biol Phys. 2012;82(5):e787–93. 10.1016/j.ijrobp.2011.11.030 22331003 PMC3285390

[3] Murray JR, Tree AC, Alexander EJ, Sohaib A, Hazell S, Thomas K, et al. Standard and hypofractionated dose escalation to intraprostatic tumor nodules in localized prostate cancer: Efficacy and toxicity in the DELINEATE trial. Int J Radiat Oncol Biol Phys. 2020;106(4):715–24. 10.1016/j.ijrobp.2019.11.402 31812718

[4] Kerkmeijer LG, Groen VH, Pos FJ, Haustermans K, Monninkhof EM, Smeenk RJ, et al. Focal boost to the intraprostatic tumor in external beam radiotherapy for patients with localized prostate cancer: results from the FLAME randomized phase III trial. 2021;39(7):787–96. 10.1200/JCO.20.02873 33471548

[5] Steenbergen P, Haustermans K, Lerut E, Oyen R, De Wever L, Van den Bergh L, et al. Prostate tumor delineation using multiparametric magnetic resonance imaging: Inter-observer variability and pathology validation. Radiother Oncol. 2015;115(2):186–90. 10.1016/j.radonc.2015.04.012 25935742

[6] van Schie MA, Dinh CV, van Houdt PJ, Pos FJ, Heijmink SW, Kerkmeijer LG, et al. Contouring of prostate tumors on multiparametric MRI: Evaluation of clinical delineations in a multicenter radiotherapy trial. Radiother Oncol. 2018;128(2):321–6. 29731160 10.1016/j.radonc.2018.04.015

[7] Fendler WP, Calais J, Eiber M, Flavell RR, Mishoe A, Feng FY, et al. Assessment of 68Ga-PSMA-11 PET accuracy in localizing recurrent prostate cancer: a prospective single-arm clinical trial. JAMA Oncol. 2019;5(6):856–63. 10.1001/jamaoncol.2019.0096 30920593 PMC6567829

[8] Liu W, Fakir H, Randhawa G, Alfano R, Corkum M, Kassam Z, et al. Defining radio-recurrent intra-prostatic target volumes using PSMA-targeted PET/CT and multi-parametric MRI. Clin Transl Radiat Oncol. 2022;32:41–7. 34841094 10.1016/j.ctro.2021.11.006 PMC8606298

[9] Matushita CS, Silva AM, Schuck PN, Bardisserotto M, Piant DB, Pereira JL, et al. 68 Ga-Prostate-specific membrane antigen (PSMA) positron emission tomography (pet) in prostate cancer: a systematic review and meta-analysis. Int Braz J Urol. 2021;47:705–29. 10.1590/s1677-5538.ibju.2019.0817 33566470 PMC8321470

[10] Zamboglou C, Fassbender TF, Steffan L, Schiller F, Fechter T, Carles M, et al. Validation of different PSMA-PET/CT-based contouring techniques for intraprostatic tumor definition using histopathology as standard of reference. Radiother Oncol. 2019;141:208–13. 10.1016/j.radonc.2019.07.002 31431386

[11] Spohn SK, Kramer M, Kiefer S, Bronsert P, Sigle A, Schultze-Seemann W, et al. Comparison of manual and semi-automatic [18F] PSMA-1007 PET based contouring techniques for intraprostatic tumor delineation in patients with primary prostate cancer and validation with histopathology as standard of reference. Front Oncol. 2020;10:600690. 10.3389/fonc.2020.600690 33365271 PMC7750498

[12] Alfano R, Bauman GS, Liu W, Thiessen JD, Rachinsky I, Pavlosky W, et al. Histologic validation of auto-contoured dominant intraprostatic lesions on [18F] DCFPyL PSMA-PET imaging. Radiother Oncol. 2020;152:34–41. 10.1016/j.radonc.2020.08.008 32827589

[13] Im H-J, Bradshaw T, Solaiyappan M, Cho SY. Current methods to define metabolic tumor volume in positron emission tomography: which one is better? Nucl Med Mol Imaging. 2018;52:5–15. 29391907 10.1007/s13139-017-0493-6 PMC5777960

[14] Gardin I. Methods to delineate tumour for radiotherapy by fluorodeoxyglucose positron emission tomography. Cancer/Radiothérapie. 2020;24(5):418–22. 10.1016/j.canrad.2020.04.008 32507519

[15] Nilsson E, Sandgren K, Grefve J, Jonsson J, Axelsson J, Lindberg AK, et al. The grade of individual prostate cancer lesions predicted by magnetic resonance imaging and positron emission tomography. Commun Med. 2023;3(1):164. 10.1038/s43856-023-00394-7 37945817 PMC10636013

[16] Sandgren K, Nilsson E, Lindberg AK, Strandberg S, Blomqvist L, Bergh A, et al. Registration of histopathology to magnetic resonance imaging of prostate cancer. Phys Imaging Radiat Oncol. 2021;18:19–25. 10.1016/j.phro.2021.03.004 34258403 PMC8254194

[17] Nyholm T, Berglund M, Brynolfsson P, Jonsson J. EP-1533: ICE-Studio-An Interactive visual research tool for image analysis. Radiother Oncol. 2015;115:S837. 10.1016/S0167-8140(15)41525-7

[18] Klein S, Staring M, Murphy K, Viergever MA, Pluim JP. Elastix: a toolbox for intensity-based medical image registration. IEEE Trans Med Imaging. 2009;29(1):196–205. 10.1016/S0167-8140(15)41525-7 19923044

[19] McCormick M, Liu X, Jomier J, Marion C, Ibanez L. ITK: enabling reproducible research and open science. Front Neuroinform. 2014;8:13. 10.3389/fninf.2014.00013 24600387 PMC3929840

[20] Warfield SK, Zou KH, Wells WM. Simultaneous truth and performance level estimation (STAPLE): an algorithm for the validation of image segmentation. IEEE Trans Med Imaging. 2004;23(7):903–21. 10.1109/TMI.2004.828354 15250643 PMC1283110

[21] Bradley D, Roth G. Adaptive thresholding using the integral image. J Graph Tools. 2007;12(2):13–21. 10.1080/2151237X.2007.10129236

[22] Lee I, Im H-J, Solaiyappan M, Cho SY. Comparison of novel multi-level Otsu (MO-PET) and conventional PET segmentation methods for measuring FDG metabolic tumor volume in patients with soft tissue sarcoma. EJNMMI Phys. 2017;4(1):1–10. 10.1186/s40658-017-0189-0 28921170 PMC5603470

[23] Zou KH, Warfield SK, Bharatha A, Tempany CM, Kaus MR, Haker SJ, et al. Statistical validation of image segmentation quality based on a spatial overlap index1: scientific reports. Acad Radiol. 2004;11(2):178–89. 14974593 10.1016/S1076-6332(03)00671-8 PMC1415224

[24] Boellaard R, Krak NC, Hoekstra OS, Lammertsma AA. Effects of noise, image resolution, and ROI definition on the accuracy of standard uptake values: a simulation study. J Nucl Med. 2004;45(9):1519–27. 15347719

[25] Foster B, Bagci U, Mansoor A, Xu Z, Mollura DJ. A review on segmentation of positron emission tomography images. Comput Biol Med. 2014;50:76–96. 24845019 10.1016/j.compbiomed.2014.04.014 PMC4060809

[26] Seifert R, Sandach P, Kersting D, Fendler WP, Hadaschik B, Herrmann K, et al. Repeatability of 68Ga-PSMA-HBED-CC PET/CT–derived total molecular tumor volume. J Nucl Med. 2022;63(5):746–53. 10.2967/jnumed.121.262528 34446454 PMC9051594

[27] Zhang Y-N, Lu Z-G, Wang S-D, Lu X, Zhu L-L, Yang X, et al. Gross tumor volume delineation in primary prostate cancer on 18F-PSMA-1007 PET/MRI and 68Ga-PSMA-11 PET/MRI. Cancer Imaging. 2022;22(1):1–11. 10.1186/s40644-022-00475-1 35869521 PMC9308314

[28] Soret M, Bacharach SL, Buvat I. Partial-volume effect in PET tumor imaging. J Nucl Med. 2007;48(6):932–45. 10.2967/jnumed.106.035774 17504879

[29] Caribé PR, Koole M, D’Asseler Y, Deller TW, Van Laere K, Vandenberghe S. NEMA NU 2–2007 performance characteristics of GE Signa integrated PET/MR for different PET isotopes. EJNMMI Phys. 2019;6:1–13. 10.1186/s40658-019-0247-x 31273558 PMC6609673

[30] Dietlein M, Kobe C, Kuhnert G, Stockter S, Fischer T, Schomäcker K, et al. Comparison of [18 F] DCFPyL and [68 Ga] Ga-PSMA-HBED-CC for PSMA-PET imaging in patients with relapsed prostate cancer. Mol Imaging Biol. 2015;17:575–84. 10.1007/s11307-015-0866-0 26013479 PMC4493776

[31] Aide N, Lasnon C, Kesner A, Levin CS, Buvat I, Iagaru A, et al. New PET technologies–embracing progress and pushing the limits. Eur J Nucl Med Mol Imaging. 2021;48(9):2711–26. 10.1007/s00259-021-05390-4 34081153 PMC8263417

