# Supplementary material for: Accuracy of gross tumour volume delineation with [68Ga]-PSMA-PET compared to histopathology for high-risk prostate cancer
Source: Acta Oncol. 2024 Jun 23;63:39041. doi: 10.2340/1651-226X.2024.39041 (PMC11332483; doi:10.2340/1651-226X.2024.39041)
Supplement: Accuracy of gross tumour volume delineation with [68Ga]-PSMA-PET compared to histopathology for high-risk prostate cancer [file AO-63-39041-s1.pdf]

Supplementary material has been published as submitted. It has not been copyedited, or typeset by Acta Oncologica

Table S.1: Patients characteristics.

Patient	Age (y)	Weight (kg)	Pre-operative PSA (ng/ml)	pT-stage	Days between imaging and surgery	Injected activity PSMA (MBq)
**P1**	68	100	12.6	3	50	181
**P2**	69	92	6.5	3	45	178
**P3**	70	84	6.4	2	5	162
**P4**	66	91	6.0	3	59	181
**P5**	64	69	13.3	2	20	138
**P6**	56	81	4.2	3	7	157
**P7**	75	91	8.4	3	24	170
**P8**	76	85	6.3	3	2	166
**P9**	56	90	6.1	2	31	180
**P10**	56	93	10.4	3	20	180
**P11**	65	91	3.9	3	23	181
**P12**	72	75	9.4	3	27	152
**P13**	59	70	4.9	3	40	143
**P14**	60	82	9.2	3	12	159
**P15**	54	77	10.9	3	19	145

Table S.2: Characteristics of Sequence Parameters: The reported values represent averages across all individuals in the subset.

	**Ax T2W**
**Echo Time (ms)**	103.2
**Repetition Time (ms)**	9582
**Flip Angle (˚)**	125
**Slice thickness (mm)**	2.5
**Matrix size**	512 × 512

Table S.3: Median lesion coverage DSC and FDR for adaptive threshold method

S **value**	**Median Lesion coverage ratio (IQR)**	**Median DSC (IQR)**	**Median FDR(IQR)**
0.1	0.84 (0.72–0.96)	0.21 (0.09–0.36)	0.85 (0.75–0.95)
0.2	0.85 (0.76–0.97)	0.21 (0.08–0.31)	0.86 (0.75–0.95)
0.3	0.90 (0.82–0.98)	0.19 (0.07–0.29)	0.88 (0.78–0.96)
0.4	0.94 (0.83–0.98)	0.16 (0.07–0.28)	0.91 (0.80–0.98)
0.5	0.98 (0.93–1.00)	0.11 (0.07–0.21)	0.94 (0.82–1.00)

Table S.4: Overview of manual GTVs in comparison with histopathology as a reference standard.

	**Median lesion coverage ratio (IQR)**	**Median measured volume in ml (IQR)**
**Oncologist 1**	0.57 (0.25–0.70)	1.46 (0.86–2.8)
**Oncologist 2**	0.50 (0.20–0.70)	1.28 (0.88–1.80)
**Oncologist 3**	0.23 (0.10–0.53)	0.82 (0.54–1.16)
**Oncologist 4**	0.42 (0.12–0.56)	1.10 (0.64–2.4)
**STAPLE algorithm**	0.47 (0.20–0.67)	1.30 (0.82–2.28)

Registration uncertainty compensation:

After the image registration process, an extra compensation was implemented to enhance overlap measures while staying within the specified registration error limit of 1.7 mm. This compensation process included aligning in-vivo GTV delineations with histology segmentations, utilizing the kappa statistic metric to maximize the Dice Similarity Coefficient (DSC) while introducing a penalty term based on translation distance. The weighting of this penalty term was changed to guarantee that the median translation distance for all patients stayed at 1.7 mm. To find the ideal weight, a combined GTV based on all MRI sequences (using the STAPLE algorithm) was utilised. Once we had determined the desired settings to achieve this specific error margin, we applied the same translation information to shift the PET-based GTVs obtained from each delineation method to the lesion annotations derived from histopathology. Table S5 shows the lesion coverage and DSC values before and after the registration uncertainty compensation process.

Table S.5: Overview of various PET-based delineation methods with considering histopathology as a reference standard, before and after the registration uncertainty process.

Methods	*Median Lesion coverage ratio (*IQR)*		*Median DSC (IQR)*	
	Before	After	Before	After
Manual	0.47 (0.21–0.67)	0.59 (0.49–0.72)	0.44 (0.24–0.58)	0.51 (0.31–0.62)
SUV>2	0.93 (0.86–0.99)	0.95 (0.93–0.99)	0.15 (0.07–0.35)	0.16 (0.08–0.35)
SUV>2.5	0.83 (0.72–0.93)	0.89 (0.78–0.96)	0.16 (0.10–0.40)	0.17 (0.14–0.41)
SUV> 3	0.72 (0.58–0.89)	0.82 (0.64–0.91)	0.26 (0.08–0.42)	0.27 (0.14–0.47)
SUV>3.5	0.61 (0.40–0.82)	0.77 (0.57–0.86)	0.36 (0.09–0.51)	0.37 (0.17–0.55)
SUV> 4	0.51 (0.23–0.74)	0.71 (0.57–0.85)	0.41 (0.14–0.54)	0.45 (0.21–0.60)
SUV>4.5	0.44 (0.21–0.65)	0.66 (0.38–0.70)	0.38 (0.12–0.55)	0.40 (0.23–0.59)
SUVmax20%	0.87 (0.62–0.97)	0.90 (0.75–0.97)	0.38 (0.11–0.54)	0.40 (0.17–0.57)
SUVmax30%	0.64 (0.44–0.73)	0.68 (0.46–0.79)	0.38 (0.10–0.50)	0.39 (0.14–0.51)
SUVmax40%	0.38 (0.24–0.62)	0.44 (0.36–0.69)	0.35 (0.10–0.46)	0.37 (0.13–0.59)
SUVmax50%	0.24 (0.13–0.40)	0.32 (0.13–0.60)	0.29 (0.09–0.43)	0.35 (0.11–0.48)
Adaptive	0.78 (0.62–0.94)	0.90 (0.73–0.92)	0.29 (0.08–0.40)	0.29 (0.11–0.46)
Otsu2	0.61 (0.45–0.85)	0.74 (0.49–0.89)	0.38 (0.1–0.52)	0.4 (0.14–0.62)
Otsu3	0.29 (0.23–0.55)	0.41 (0.25–0.64)	0.35 (0.08–0.5)	0.39 (0.16–0.59)
